# Kurarinol A from *Sophora flavescens* suppresses hepatic stellate cell activation via the TLR4/MyD88/NF-*κ*B pathway

**DOI:** 10.3389/fphar.2026.1948257

**Published:** 2026-10-22

**Authors:** Xing-Jun Chen, Qian Wang, Mu-Zhen Wang, Xu Zhang, Shang-Gao Liao, Zhu Zeng, Xiao-Peng Li, Lihui Wang, Bo Tu, Yan Lin

**Affiliations:** 1 State Key Laboratory of Discovery and Utilization of Functional Components in Traditional Chinese Medicine and School of Pharmaceutical Sciences, Guizhou Medical University, Guiyang, Guizhou, China; 2 Engineering Research Center for the Prevention and Treatment of Chronic Diseases by Authentic Medicinal Materials, Guizhou Provincial Department of Education, Guizhou Medical University, Guiyang, Guizhou, China; 3 Key Laboratory of Quality Control and Evaluation of Traditional Chinese and Ethnic Medicines, Administration for Market Regulation of Guizhou Province, Guiyang, Guizhou, China; 4 Guiyang Longmaster Information Technology Co, Ltd., Guiyang, Guizhou, China; 5 College of Computer Science and Technology, Guizhou University, Guiyang, Guizhou, China

**Keywords:** kurarinol A (KA), liver fibrosis, MyD88, *Sophora flavescens*, TLR4/NF-*κ*B pathway

## Abstract

**Introduction:**

*Sophora flavescens* is traditionally used in TCM and Miao/Mongolian/Yi ethnomedicines for hepatobiliary disorders, yet its bioactive flavonoid constituents and mechanisms remain incompletely understood. This study investigated the anti-hepatic fibrosis efficacy, mechanism, and target of the ethyl acetate fraction (EAF) from *S. flavescens* and its key lavandulyl flavonoid kurarinol A (KA).

**Methods:**

A CCl4-induced mouse hepatic fibrosis model was established to evaluate liver injury and fibrosis by H&E/Masson staining and detection of serum ALT/AST and hepatic α-SMA/fibronectin/Hyp. A TGF-β1-activated LX-2 cell model was established, with cell viability, migration, and apoptosis evaluated by MTS, wound-healing, and Annexin V-FITC/PI assays. Transcriptomics, KEGG analysis, molecular docking, Molecular dynamics (MD) simulation, CETSA, and MyD88 knockdown/overexpression assays were applied to explore the underlying mechanism.

**Results:**

*In vivo*, EAF (50 and 100 mg/kg) alleviated CCl4-induced liver injury and fibrosis and decreased the levels of fibrotic serum biomarkers. *In vitro*, KA significantly inhibited LX-2 cell activation with an IC50 of 12.65 μM (n = 3), suppressing cell proliferation and migration while inducing apoptosis. KA’s anti-fibrotic activity was validated only *in vitro*, and its contribution to the *in vivo* efficacy of the EAF remains inferred rather than directly proven. Mechanistically, KA downregulated key signaling molecules of the TLR4/NF-*κ*B pathway, thereby blocking pathway activation. Molecular docking, MD simulations and CETSA supported a direct interaction between KA and MyD88. MyD88 knockdown and overexpression assays further supported that MyD88 serves as a candidate molecular target mediating KA’s anti-fibrotic activity.

**Conclusion:**

The EAF exerts anti-fibrotic effects in vivo, and KA exhibits consistent anti-fibrotic activity *in vitro*. These findings support MyD88 as a potential molecular target mediating KA’s *in vitro* anti-fibrotic activity via TLR4/NF-*κ*B signaling. These findings not only validate the traditional use of *S. flavescens* in hepatobiliary disorders, but also offer a promising MyD88-targeting candidate warranting lead optimization for anti-fibrotic drug development.

## 1 Introduction

As a key metabolic and detoxification organ of the body, the liver plays an essential role in maintaining systemic homeostasis (Bataller and Brenner, 2005). Upon persistent injury, the liver undergoes pathological repair processes that result in fibrogenesis. Liver fibrosis is characterized by excessive deposition of extracellular matrix (ECM). Without intervention, it can progress to life-threatening cirrhosis and even hepatocellular carcinoma (Gilgenkrantz et al., 2025). Viral infection, long-term alcohol exposure, metabolic diseases (such as non-alcoholic fatty liver disease) and genetic susceptibility can drive persistent liver injury and initiate abnormal repair responses characterized by excessive activation of hepatic stellate cells (HSCs) (Tsuchiya et al., 2023; Breitkopf-He et al., 2024; Zhang L. et al., 2026; Tsuchida and Friedman, 2017). Resting HSCs are responsible for regulating the metabolic balance of the ECM. Still, under the stimulation of continuous liver injury, they are transformed into a myofibroblast phenotype with active proliferation and increased ECM secretion, which mainly drives fibrosis through the TGF-β pathway (Hernandez-Gea and Friedman, 2011). Numerous studies have suggested that hepatic stellate cell (HSC) proliferation is a central event in the progression of liver fibrosis. Consequently, elucidating the mechanisms that inhibit HSC proliferation is of great significance for developing anti-fibrotic therapies (Schwabe and Brenner, 2025; Higashi et al., 2017). TGF-β, as a core pro-fibrotic cytokine, not only directly activates HSCs but also inhibits ECM degradation by up-regulating tissue inhibitors of metalloproteinases (TIMPs), thereby forming an imbalance that is conducive to fibrosis (Issa et al., 2001; Chen et al., 2023; Yoshiji et al., 2000).

Concurrently, lipopolysaccharide (LPS) activates Toll-like receptor 4 (TLR4) signaling, which further aggravates fibrosis by amplifying the inflammatory cascade and cross-activating the TGF-β pathway in HSCs (Zou et al., 2024; Seki et al., 2007). Liver fibrosis develops as a combined consequence of profibrotic phenotypic switching in hepatic stellate cells and persistent hepatic inflammatory stimulation. In this work, we set up two cell-based models to characterize KA’s anti-fibrotic effects and explore its molecular basis. TGF-β1 stimulation produced a classic HSC activation model for phenotypic and functional evaluation, while LPS treatment was used to activate TLR4/MyD88 signaling for mechanistic studies. Although TGF-β1 is the major cytokine responsible for HSC profibrotic activation, it does not effectively drive TLR4-MyD88-mediated inflammatory signaling. We thus chose these two stimuli: TGF-β1 helps assess KA-driven anti-fibrotic functional phenotypes, and LPS lets us specifically examine the TLR4/MyD88-NF-*κ*B signaling cascade. We have previously demonstrated that the lavandulyl flavonoid kurarinol A (KA), a major constituent of *Sophora flavescens*, inhibits hepatic stellate cell activation and hepatic fibrosis through the TGF-β/Smad signaling pathway (Yang et al., 2025). In the present study, we sought to determine whether KA additionally modulates the TLR4/MyD88/NF-*κ*B pathway and to evaluate MyD88 as a potential molecular target of KA.

Myeloid differentiation factor 88 (MyD88), as a core adaptor protein of TLR4 and IL-1R signaling pathways, is a key node that drives NF-*κ*B and MAPK-mediated pro-inflammatory and pro-fibrotic responses (Zhang et al., 2022; Zhang et al., 2024; Tang et al., 2023). Accumulating pharmacological evidence has suggested that targeting the TLR4/MyD88 axis effectively alleviates hepatic fibrosis in preclinical models, represented by alantolactone-based pathway inhibition (Zhang X. et al., 2026; Almukadi et al., 2025). As a critical convergence node of multiple inflammatory cascades, MyD88 has emerged as a promising therapeutic target for liver fibrosis. However, the only anti-hepatic fibrosis candidate drug TJ-M2010-5, that has entered clinical trials has certain limitations in drug-likeness and safety, which highlights the urgent need to develop new therapeutic drugs for this signaling cascade (Chen et al., 2020).

*Sophora flavescens* Ait. (Fabaceae) is a traditional Chinese medicine commonly used in Chinese medicine. It is first recorded in Shennong’s Classic of Materia Medica. It is described as bitter in taste and cold in nature, acting on the heart, liver, stomach, large intestine, and bladder meridians. This herbal medicine has traditionally been used to clear heat, eliminate dampness, kill parasites, and promote diuresis. Clinically, it can be indicated for conditions including heat-induced dysentery, hematochezia, jaundice with anuria, leukorrhea, genital swelling and pruritus, eczema, damp sores, cutaneous pruritus, scabies, leprosy, and trichomonal vaginitis (Wang and Lan, 2025). The ancient Chinese herbal work Mingyi Bielu additionally records that it can nourish liver and gallbladder qi, tonify the middle energizer, and improve eyesight. Beyond its long-standing use in mainstream Chinese medicine, *S. flavescens* has also been employed by several Chinese ethnic groups. In Miao medicine of Guizhou, the root (Miao names: “jab gongx saib” in Qiandongnan, “vuab ginl rongdlib” in Qiannan, “faik kend” in Bijie, and “yes ninb”) has been used as an antipyretic and anti-diarrheal remedy against febrile diarrheal conditions, whole-body pruritic wind-type skin diseases, and itching, and has also been recorded for dysentery, jaundice, skin itching, and sores (Chinese Materia Medica Editorial Board, 2005). In Mongolian medicine, the root (“Dagule-wubusi” and “Daogule-ebusu”) is recorded for the treatment of epidemic febrile diseases, jaundice, dysentery, damp-heat, rheumatic complaints, brucellosis, skin itching, and sores, and for external application against vulvar itching (Chinese Materia Medica Editorial Board, 2005). In Yi medicine, the whole plant is documented for treating liver–gallbladder damp-heat, dysentery, sores, hemorrhoid–fistula–eczema conditions, and pruritus (Chinese Materia Medica Editorial Board, 2005). These ethnomedicinal records consistently associate *S. flavescens* with hepatobiliary, inflammatory, and pruritic conditions, providing a supportive traditional-use basis for investigating its anti-hepatic-fibrosis activity. Modern pharmacological studies have shown that alkaloids such as matrine and oxymatrine in *S*. *flavescens* show good activity in liver protection (Zhang et al., 2001). Total flavonoids of *S*. *flavescens* also have an improvement effect on nonalcoholic fatty liver disease (Gu et al., 2025). Accumulating evidence has suggested that *S. flavescens* extracts exert prominent anti-inflammatory and hepatoprotective effects (Kong et al., 2024; Yang et al., 2024). Nevertheless, the specific functional ingredients and detailed molecular mechanisms remain unclear (Jia et al., 2026; Wen et al., 2026). Against this background, we successfully identified kurarinol A (KA), a rare lavandulyl flavonoid from *S. flavescens*, as a promising candidate compound for ameliorating liver injury.

The tracking of active components of natural products usually follows the classical paradigm of “crude extract activity screening → active fraction localization → monomer compound mechanism analysis.” Consistent with this paradigm, our previous work confirmed the preliminary hepatoprotective effect of KA (Lin et al., 2023). Considering that crude herbal fractions exert holistic *in vivo* efficacy through multi-component synergy while pure monomers are suitable for precise *in vitro* mechanistic exploration, the present study adopted a rigorous hierarchical design: the ethyl acetate fraction (EAF) of *S*. *flavescens* was used for *in vivo* anti-fibrotic evaluation in CCl_4_-induced mice, whereas purified KA was applied for *in vitro* functional and mechanistic studies in TGF-β1-stimulated LX-2 cells. Herein, we propose a clear, testable hypothesis that KA directly binds to MyD88 to suppress TLR4/NF-*κ*B signaling and consequently inhibits HSC activation to produce its *in vitro* anti-hepatic fibrosis activity. This study aimed to verify the *in vivo* antifibrotic efficacy of the ethyl acetate fraction from *S*. *flavescens*, characterize the *in vitro* antifibrotic activity of KA, and systematically validate MyD88 as KA’s core molecular target through transcriptomics, molecular simulation, and genetic manipulation assays.

We hypothesized that KA exerts anti-fibrotic activity via direct binding to MyD88, thereby inhibiting downstream TLR4/NF-*κ*B-dependent inflammatory-fibrotic signaling. To test this hypothesis, the present study aimed: (1) to evaluate the anti-fibrotic effects of S. flavescens EAF and KA in CCl_4_-injured mice and activated HSCs; (2) to validate MyD88 as the direct molecular target of KA; (3) to characterize the downstream signaling changes mediated by KA-MyD88 interaction. The core innovation of this study is to clarify the MyD88-dependent anti-fibrotic mechanism of KA for the first time, providing a novel natural candidate and mechanistic basis for MyD88-targeted anti-fibrotic drug development.

## 2 Materials and methods

### 2.1 Reagents and chemicals

The dried root of *S. flavescens* was purchased in December 2022 from Changzhi County, Changzhi City, Shanxi Province, China, and was identified by Professor Long Qingde as the dried root of *S. flavescens* Ait. (Fabaceae). A voucher specimen of the material used in this study (No. GMU202212) has been deposited in the Herbarium of Traditional Chinese Medicine and Ethnic Medicine at Guizhou Medical University, ensuring proper curation and accessibility of the specimen. An appropriate amount of dried roots was crushed and extracted with 90% ethanol for three times, 2 h each time. The extracts were combined and concentrated under reduced pressure until no alcohol taste was detected. The concentrated solution was dispersed with water, and then extracted with petroleum ether and ethyl acetate in turn. The ethyl acetate fraction (EAF) was obtained by collecting the ethyl acetate layer and concentrating it to dryness under reduced pressure. EAF was stored at −20 °C for later use. The required concentration was prepared with a 0.5% sodium carboxymethyl cellulose (CMC-Na) solution before use. Kurarinol A (KA) was isolated and purified from the EAF in our laboratory as described in our companion chemical study of the same batch of plant material (Wang et al., 2026), in which it was reported as compound 6. Its identity was confirmed by comparison of its spectroscopic data with those reported for KA (Li et al., 2021), representative spectra were reproduced in Supplementary Figures S5, S6.

#### 2.1.1 Cell culture reagents

DMEM medium, penicillin-streptomycin, 0.25% trypsin-EDTA, dimethyl sulfoxide (DMSO) (Gibco, Beijing, China), fetal bovine serum (FBS) (Procell, Wuhan, China).

#### 2.1.2 Detection kit and biochemical reagents

MTS cell proliferation assay kit (Promega, United States); BCA protein quantification kit, phosphatase/protease inhibitor mixture, enhanced chemiluminescence substrate (ECL Plus), RIPA lysate (Solarbio, Beijing, China), total RNA extraction kit, FastKing genomic DNA removal reverse transcription premix (Tiangen, Beijing, China); TB Green Premix Ex Taq II (TaKaRa, Dalian, China), ALT, AST and hydroxyproline (Hyp) detection kits (Nanjing Jiancheng, Nanjing, China), PCR primers (Sangon, Shanghai, China).

Cytokines and inhibitors: recombinant human TGF-β1, silibinin, TGF-β1 pathway inhibitor SB431542 (Absin, Shanghai, China).

#### 2.1.3 Antibody

anti-GAPDH, Fibronectin, α-SMA, Collagen I, TLR4, MyD88, TAK1, p-p65 (Ser536), total p65, TNF-α and IL-1β primary antibody, HRP-conjugated goat anti-rabbit IgG secondary antibody (Proteintech, Wuhan, China).

### 2.2 Animal experiment

Animals: SPF male C57BL/6 mice, 6–8 weeks old. Weight 18∼22 g, provided by the Experimental Animal Center of Guizhou Medical University. Mice were fed in a constant temperature and humidity environment (22 °C ± 2 °C, 50% ± 10% RH), 12 h light/12 h dark cycle, free to eat and drink. The experiment was started after 1 week of adaptive feeding. Only male mice consistent with previous studies of CCl_4_-induced liver fibrosis (Ernst et al., 2020).

Modeling and administration: Mice were randomly divided into five groups (n = 8): normal control group, model group. Positive drug group (silybin, 100 mg/kg), EAF low dose group (50 mg/kg) and EAF high dose group (100 mg/kg). Except for the normal control group, the other four groups were intraperitoneally 10% CCl4 in olive oil (5 mL/kg, i.p., twice a week for 2 weeks). After modeling, different groups were given corresponding drugs by gavage: the normal control group and model group were given 0.5% CMC-Na solution, the positive control group was given silibinin (100 mg/kg), EAF low- and high-dose groups were given the corresponding concentration of *S*. *flavescens* EAF once a day for 2 weeks (weeks 2–4).

Randomization, blinding, and exclusion criteria. Group allocation was performed by simple random assignment. Histopathological evaluation and quantitative image analysis were conducted by two independent investigators blinded to group allocation. Animals that died during the experimental period or developed severe distress (e.g., >20% body weight loss relative to the initial body weight, inability to eat or drink, or severe injection-site infection) were recorded and, where necessary, humanely euthanized; such animals were excluded from the statistical analysis. At the end of the experiment, mice were deeply anesthetized, and blood samples were collected by retro-orbital bleeding. Immediately afterward, mice were humanely euthanized, and liver tissues were harvested for subsequent analyses.

### 2.3 Ethics statement

All animal experiments were reviewed and approved by the Experimental Animal Ethics Committee of Guizhou Medical University (approval No. 2100066) and were conducted in accordance with the ARRIVE 2.0 guidelines and the 3R principle (Replacement, Reduction, Refinement). Mice were housed in the specific pathogen-free (SPF) facility of the Laboratory Animal Center of Guizhou Medical University (animal use license No. SYXK (Gui) 2023-0002) under controlled environmental conditions, and all efforts were made to minimize animal suffering and the number of animals used.

### 2.4 Histological analyses

Liver tissue specimens were processed by paraffin embedding, followed by sectioning and drying. The pathological alterations and collagen deposition within the liver tissue were evaluated using hematoxylin-eosin (H&E) staining and Masson staining techniques. Histopathological evaluation of H&E− and Masson-stained liver sections was performed by an experienced investigator blinded to group allocation. Collagen deposition in Masson-stained sections was quantified by computer-assisted image analysis (ImageJ) and expressed as the percentage of collagen-positive area relative to total section area.

### 2.5 Cell culture, proliferation, migration, and apoptosis assays

The LX-2 cell line was purchased from Wuhan Procell Life Science and Technology Co., Ltd. Cells were cultured in DMEM containing 10% FBS and 1% penicillin-streptomycin at 37 °C, 5% CO_2_ and in humidified incubators.

Cell proliferation activity was determined by the MTS assay. LX-2 cells were seeded in 96-well plates (4 × 10^3^ cells/well), cultured for 24 h to about 80% confluence, and then stimulated with 10 ng/mL TGF-β1 (DMEM containing 2% FBS) for 24 h to induce activation. Subsequently, the compound to be tested (40 μM), the EAF (25 μg/mL), or the positive control silibinin (diluted with DMEM containing 2% FBS) was added for 24 h. 10 μL MTS reagent was added to each well, and the absorbance was measured at 490 nm after incubation for 3 h. A single concentration (40 μM for pure compounds; 25 μg/mL for the EAF) was used for the initial activity screen to rank the 35 compounds, active hits were subsequently evaluated by full dose–response analysis. Because the EAF is a multicomponent mixture, the mass-based concentration (25 μg/mL) is not directly comparable to the molar concentration used for pure compounds; the screen therefore serves as a qualitative activity ranking rather than a quantitative potency comparison.

Cell migration ability was evaluated by scratch test. LX-2 cells were seeded in 6-well plates (8 × 10^4^ cells/well), cultured to monolayer fusion, scratched with a sterile pipette tip, and washed with PBS to remove exfoliated cells. The scratch area was measured by ImageJ software, and the mobility was calculated: mobility (%) = [ W (0 h) − W (24 h/48 h)]/W (0 h) × 100%.

#### 2.5.1 Apoptosis detection

Annexin V-FITC/PI double staining flow cytometry was used. LX-2 cells were stimulated with TGF-β1 (10 ng/mL) for 24 h, and then treated with KA (2.5, 5, 10 μM) or silibinin (50 μM) for 24 h. The cells were collected and stained with Annexin V-FITC/PI for 15 min, and the apoptosis rate was detected by flow cytometry. Early-apoptotic (Annexin V^+^ PI^−^) and late-apoptotic (Annexin V^+^ PI^+^) cell populations were summed to calculate the total apoptosis rate. Necrotic cells and cellular debris were excluded from this calculation.

### 2.6 RT-qPCR analysis

After the drug treatment described in the LX-2 cell scratch assay section, total RNA was isolated for quantitative real-time PCR (RT-qPCR). Briefly, LX-2 cells in the logarithmic growth phase were treated according to experimental groups and cultured for 24 h. The culture medium was discarded, and cells were washed twice with PBS. TRIzol reagent was added to lyse cells, and total RNA was extracted following the manufacturer’s instructions. RNA concentration and purity were determined using a NanoDrop 2000 spectrophotometer. One microgram of total RNA was used for cDNA synthesis with the FastKing gDNA Removal RT SuperMix. qPCR amplification was carried out on an ABI 7500 system using TB Green Premix Ex Taq II. With GAPDH as the internal reference, the relative expression was calculated by the 2^^−ΔΔCt^ method. The primer sequence is shown in Table 1.

**TABLE 1:** Primer sequences used for RT-qPCR analysis.

Gene	Forward	Reverse
*GAPDH*	GGA​GTC​CAC​TGG​CGT​CTT​CAC	GCT​GAT​GAT​CTT​GAG​GCT​GTT​GTC
*Collagen I*	GCC​TCT​GCT​CTC​CGA​CCT​CTC	GCT​TTG​TGC​TTT​GGG​AAG​TTG​TCT​C
*α-SMA*	CTT​CGT​TAC​TAC​TGC​TGA​GCG​TGA​G	CCC​ATC​AGG​CAA​CTC​GTA​ACT​CTT​C
*Fibronectin*	ACG​ACT​GTG​GAC​CAA​GTT​GAT​GAC	CTG​TGC​TGC​TAC​CTT​CTA​CTG​ATG​G
*TLR4*	GCT​CTT​GGT​GGA​AGT​TGA​ACG​AAT​G	CAA​GCA​CAC​TGA​GGA​CCG​ACA​C
*MyD88*	GCC​GCC​TGT​CTC​TGT​TCT​TG	GTC​CGC​TTG​TGT​CTC​CAG​TTG
*TRAF6*	TGT​GCC​TCT​GCT​CTT​ACT​GTC​TG	GAA​CTA​TGG​TCA​CTG​CTG​CTT​GG
*TIRAP*	GGC​AAG​ATG​GCT​GAC​TGG​TTC	GGT​AGT​GGG​CTG​TCC​TGT​GAG
*TAK1*	AGA​CAA​TGG​AAT​TAG​GGC​AGG​TTT​G	GAC​GGA​AGG​CAG​GCA​ACA​ATC
*NF-κB*	ATG​GTG​GTC​GGC​TTC​GCA​AAC	CGC​CTC​TGT​CAT​TCG​TGC​TTC​C
*TNF-α*	AAG​GAC​ACC​ATG​AGC​ACT​GAA​AGC	AGG​AAG​GAG​AAG​AGG​CTG​AGG​AAC
*IL-1β*	GGA​CAG​GAT​ATG​GAG​CAA​CAA​GTG​G	CAA​CAC​GCA​GGA​CAG​GTA​CAG​ATT​C

### 2.7 Western blotting

Following the drug treatments described in the scratch assay section, the total protein was extracted from LX-2 cells with RIPA lysate, and the protein concentration was determined by BCA method. GAPDH was used as the internal reference for the immunoblotting analyses in Figures 1–4, whereas β-actin served as the loading control for Figure 5, as indicated in each figure legend. All subsequent immunoblotting procedures were performed as previously reported (Lin et al., 2023).

**FIGURE 1:**
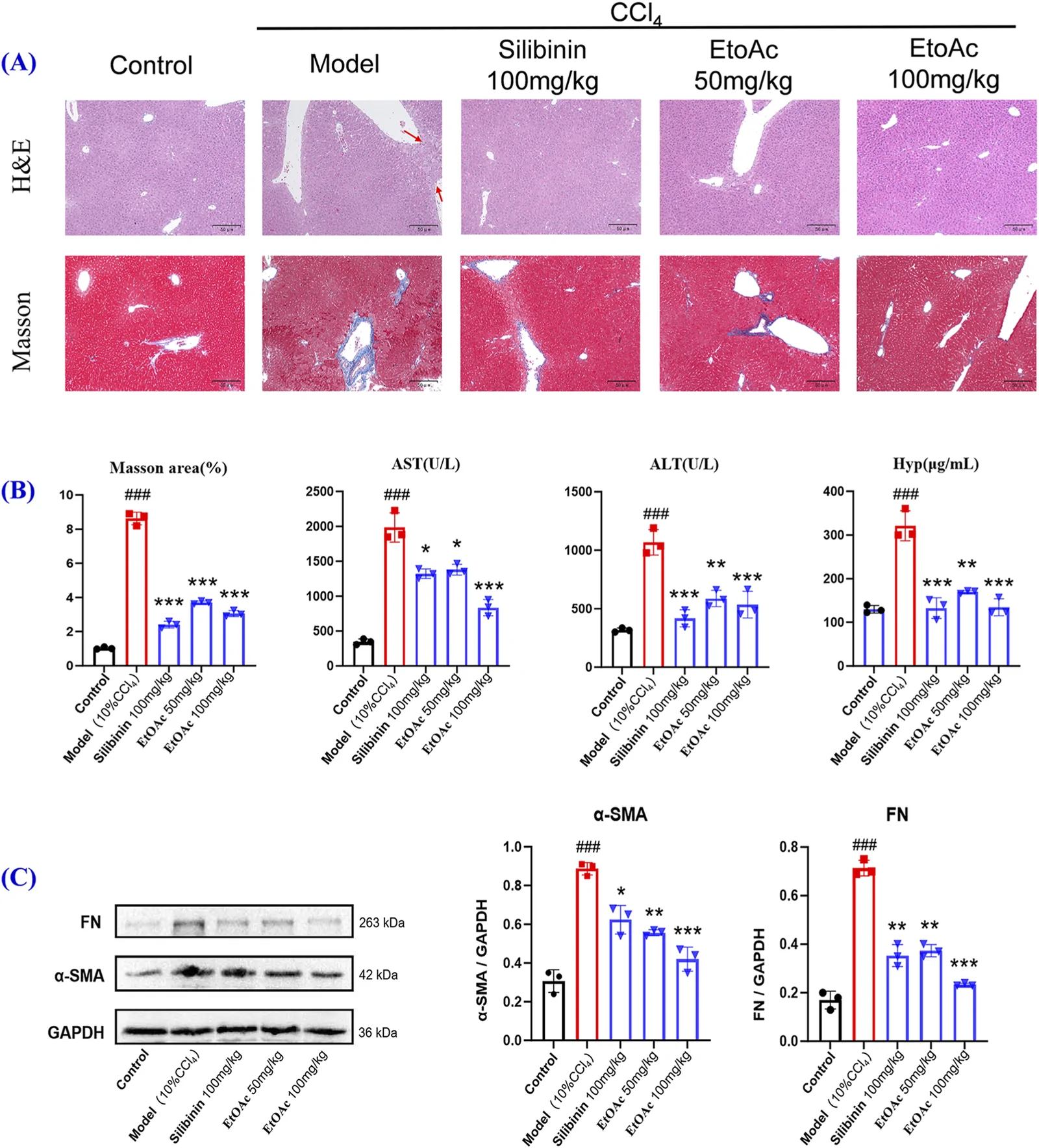
Effects of the EAF from *Sophora flavescens* on CCl_4_-induced hepatic fibrosis in mice. **(A)** Hepatic histopathology (H&E staining, 100×) and collagen accumulation (Masson staining, 100×). **(B)** Quantitative analysis of Masson-stained collagen area (%), serum AST, ALT, and hepatic Hyp. Data shown as mean ± SD (n = 8 mice per group). Statistical significance among the five groups was determined by one-way ANOVA followed by Tukey’s *post hoc* test: *P < 0.05, **P < 0.01, ***P < 0.001 vs. model controls (silibinin as positive reference). **(C)** Protein expression levels of α-SMA and FN determined by Western blot (n = 3). Densitometric data were analyzed by one-way ANOVA followed by Tukey’s *post hoc* test: ###P < 0.001 vs. normal control; *P < 0.05, **P < 0.01, ***P < 0.001 vs. model.

**FIGURE 2:**
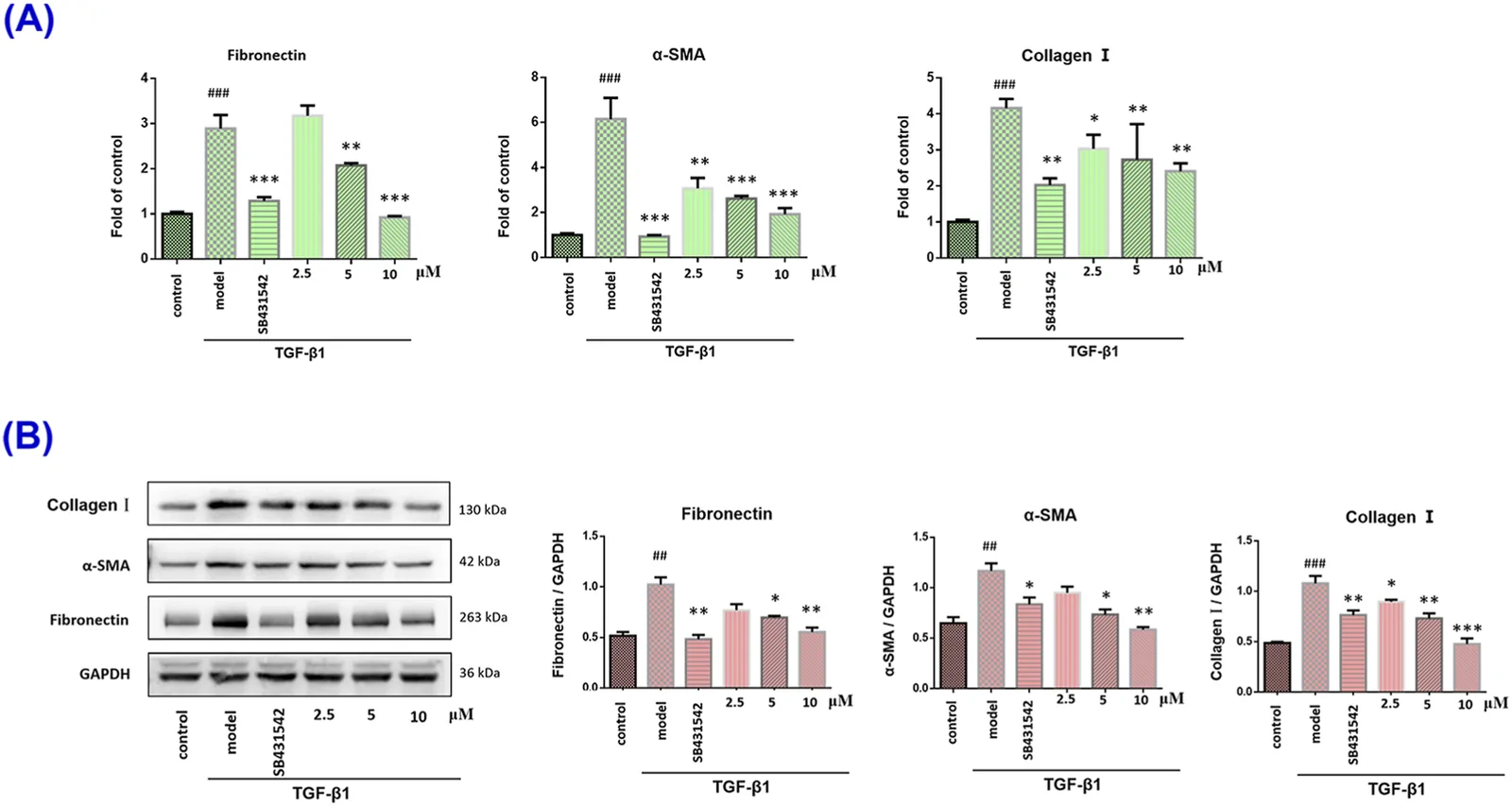
KA reduces fibronectin, α-SMA, and collagen I expression in TGF-β1-activated LX-2 cells. **(A)** RT-qPCR analysis of mRNA levels. **(B)** Western blot analysis of protein levels. SB431542 (10 µM) served as the positive control. Data are presented as mean ± SD from three independent experiments. Statistical significance was determined by one-way ANOVA followed by Tukey’s *post hoc* test: ^##^ *P* < 0.01 and ^###^ *P* < 0.001 vs. control; *P < 0.1, **P < 0.01, and ***P < 0.001 vs. the TGF-β1 model group.

**FIGURE 3:**
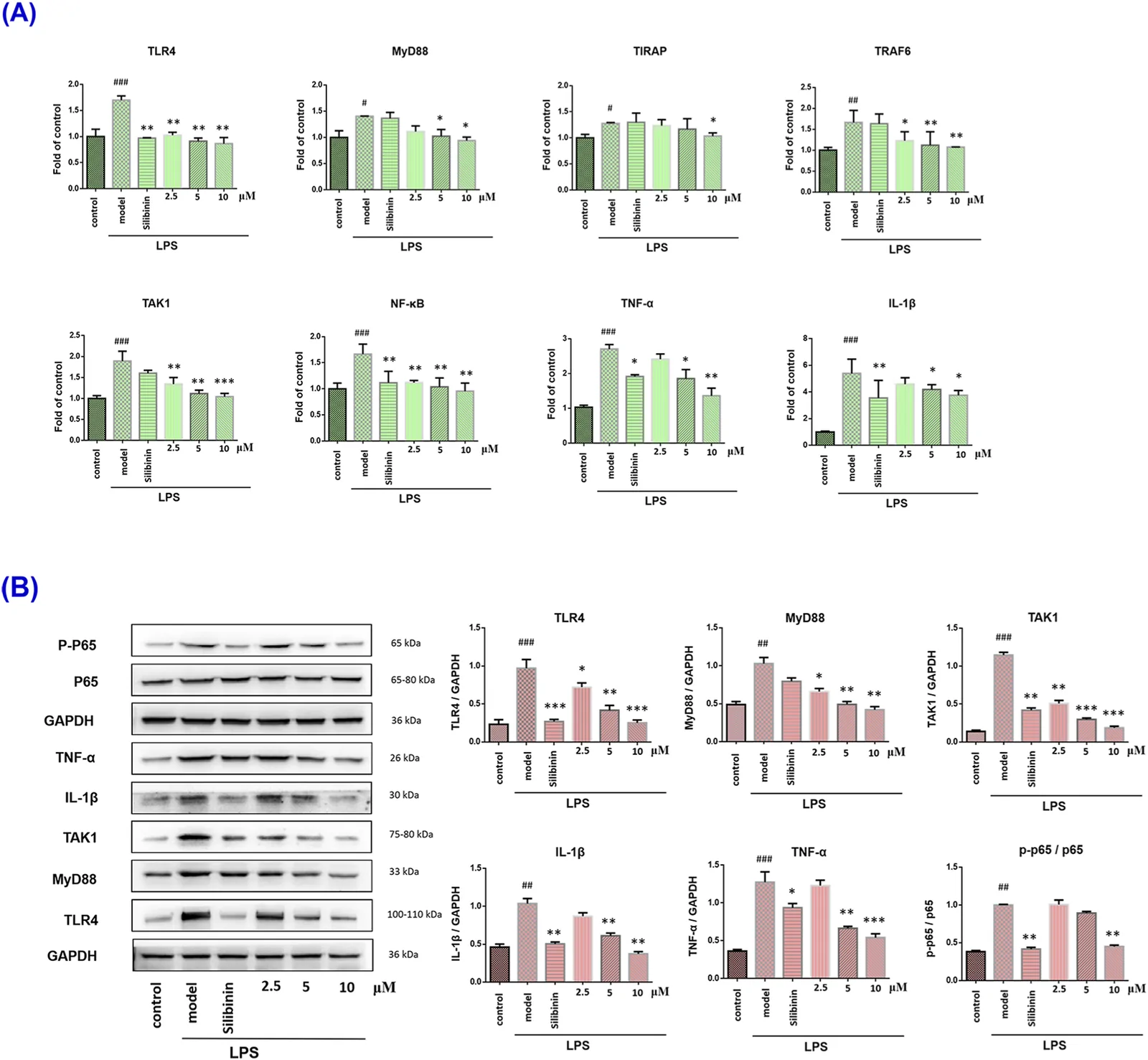
KA inhibited LPS-induced TLR4/NF-*κ*B pathway activation. **(A)** mRNA expression of *TLR4, MyD88, TIRAP, TRAF6, TAK1, NF-κB, TNF-α* and *IL-1β*. **(B)** Protein expression of TLR4, MyD88, TAK1, p-p65/p65, TNF-α and IL-1β. Silibinin (100 μM) served as the positive control. Data are presented as mean ± SD (n = 3). Statistical significance was determined by one-way ANOVA followed by Tukey’s *post hoc* test: ^#^ *P* < 0.1, ^##^ *P* < 0.01 and ^###^ *P* < 0.001 vs. control, *P < 0.1, **P < 0.01 and ***P < 0.001 vs. the LPS model group.

**FIGURE 4:**
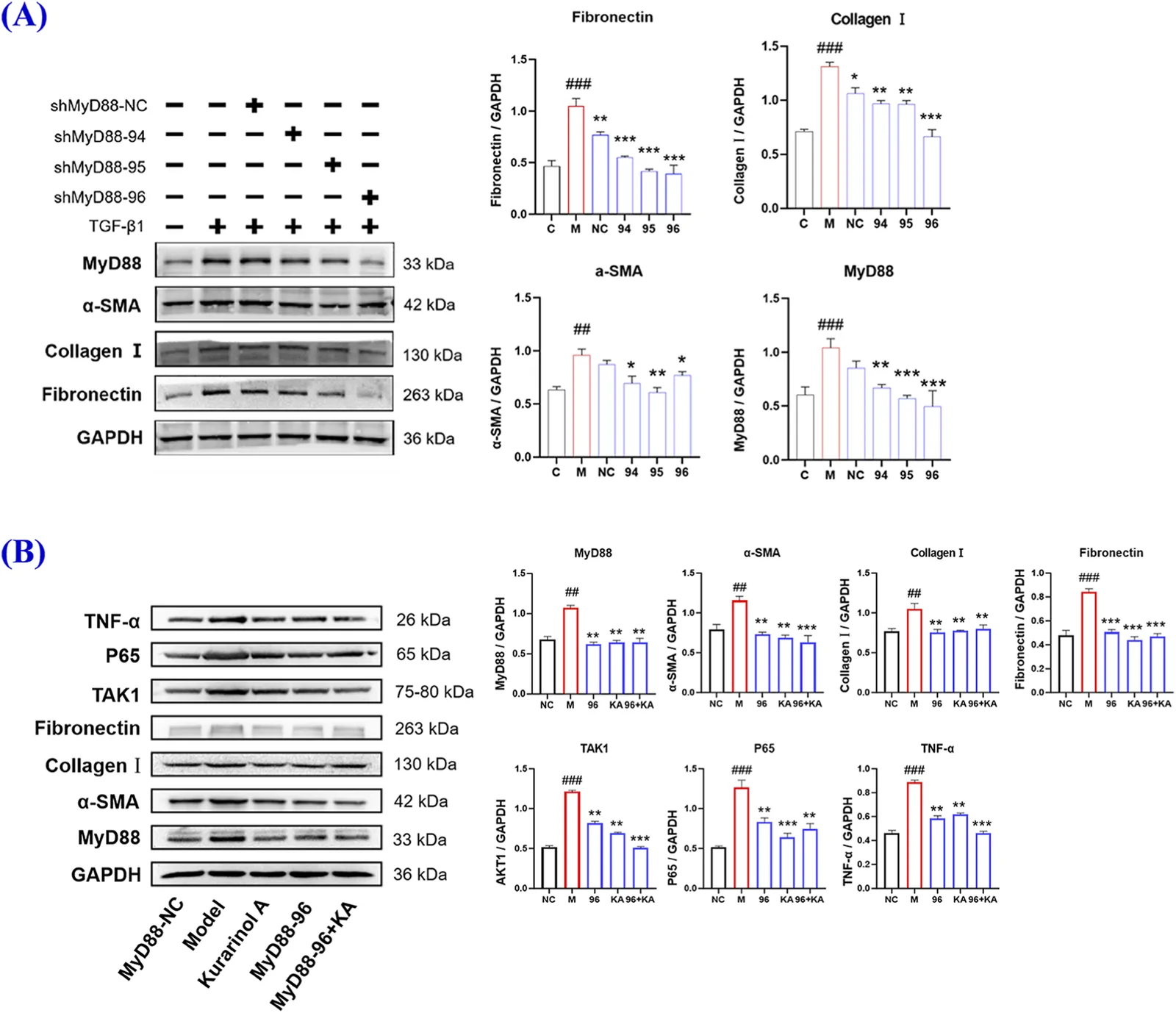
Western blot analysis of MyD88 knockdown experiments in LX-2 cells. **(A)** Screening of MyD88 knockdown viral strains (shMyD88-94, 95, 96) and their impacts on hepatic fibrosis marker expression; GAPDH: loading control. **(B)** Effects of kurarinol A (KA) on downstream protein and hepatic fibrosis marker expression in MyD88-knockdown LX-2 cells; GAPDH: loading control. For all panels, data are presented as mean ± SD (n = 3 independent experiments). Statistical significance was determined by one-way ANOVA followed by Tukey’s *post hoc* test: ^##^ *P* < 0.01, ^###^ *P* < 0.001 vs. control; *P < 0.05, **P < 0.01, ***P < 0.001 vs. model.

**FIGURE 5:**
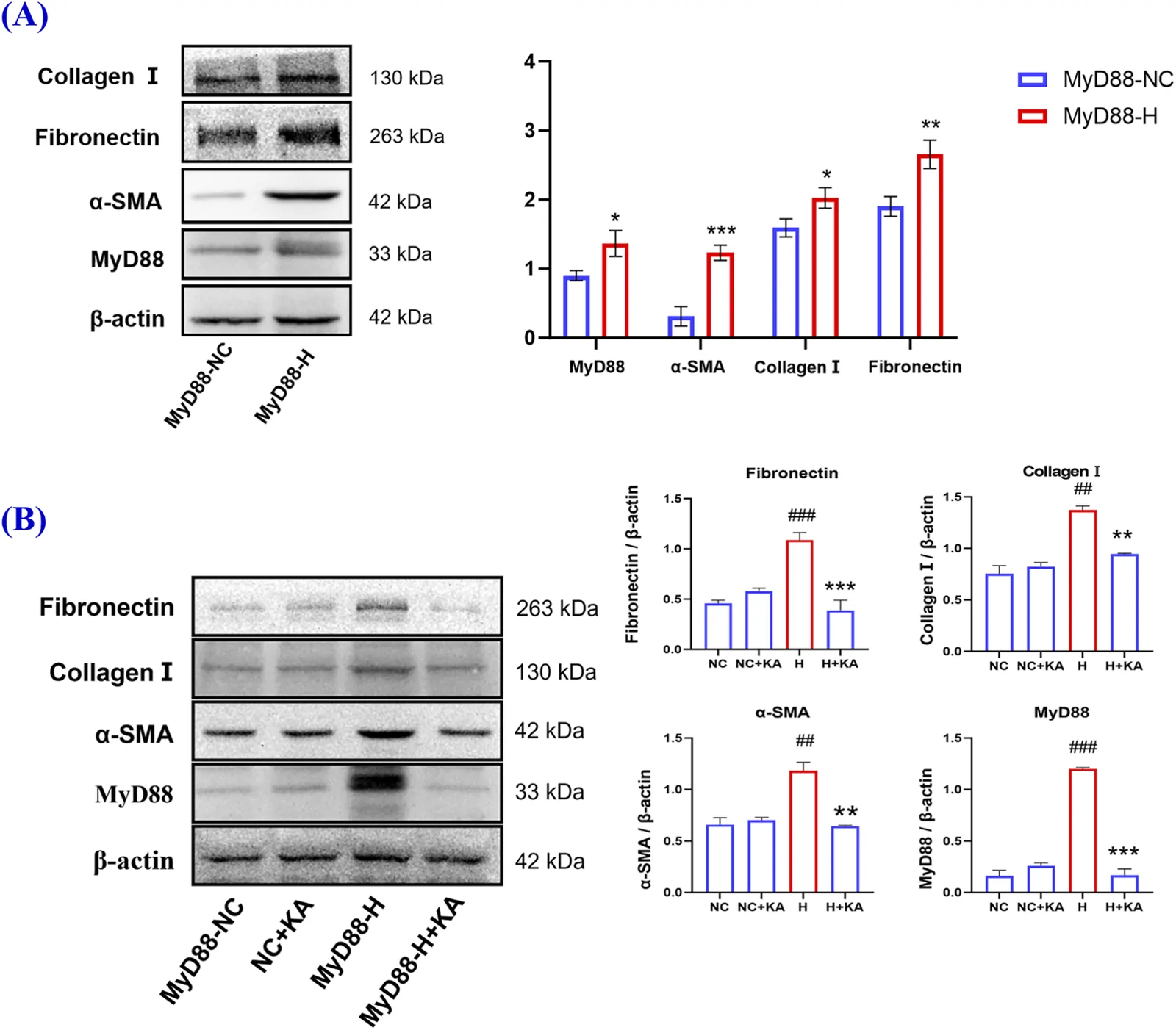
Western blot analysis of MyD88 overexpression experiments in LX-2 cells. **(A)** Western blot analysis of MyD88 overexpression (MyD88-H) and its effects on hepatic fibrosis marker expression. β-actin: loading control. Right panel: quantification of these proteins in the control (MyD88-NC) and overexpression (MyD88-H) groups. Statistical significance was determined by the unpaired two-tailed Student’s t-test; n = 3 independent biological replicates per group (*P < 0.05, **P < 0.01, ***P < 0.001 vs. MyD88-NC). **(B)** Effect of KA on the above fibrosis markers in four groups: MyD88-NC, NC + KA, MyD88-H, and H + KA. β-actin: loading control. Quantification of each target protein is shown below the blots. Differences among the four groups were analyzed by one-way ANOVA followed by Tukey’s multiple comparison test (n = 3): ^##^ *P* < 0.01 and ^###^ *P* < 0.001 vs. MyD88-NC; **P < 0.01 and ***P < 0.001 vs. MyD88-H.

### 2.8 Transcriptome sequencing

To characterize gene expression shifts linked to hepatic stellate cell activation, we cultured LX-2 cells under three experimental conditions: untreated control, disease model, and treatment with 10 μM KA. Each group included four biological replicates, with each sample seeded at 2 × 10^6^ cells. After total RNA was extracted by the TRIzol method, the Agilent 2100 Bioanalyzer was used to assess RNA integrity and ensure that RNA quality met the requirements for database construction.

The library was constructed according to the instructions of the NEBNext Ultra RNA Library Prep Kit for Illumina. The specific steps are as follows: The purified mRNA was reverse-transcribed into cDNA after fragmentation, and the resulting cDNA was subjected to terminal repair and ligated to a sequencing adaptor. The product was purified by AMPure XP magnetic beads and enriched. After the quality inspection of the library was completed, the double-end sequencing was performed on the Illumina NovaSeq 6000 platform with a reading length of 150 bp.

The raw data after sequencing was first subjected to quality trimming by Cutadapt to remove low-quality bases and residual adapter sequences. Subsequently, the clean sequence was aligned to the human reference genome GRCh38 using HISAT2. Transcript expression was quantified by StringTie and expressed as FPKM. Differentially expressed genes were identified using DESeq2 with thresholds of |log_2_FC| > 1 and FDR <0.05. Finally, GO function annotation and KEGG pathway enrichment analysis were performed on the screened differential genes with the help of DAVID and GOseq tools.

### 2.9 Molecular docking and molecular dynamics simulations

For molecular docking, the NMR solution structure of the human MyD88 TIR domain (PDB ID: 2Z5V; first conformer) was retrieved from the Protein Data Bank. Water molecules and native ligands were removed and the protein was prepared with the Schrödinger Protein Preparation Wizard; KA was prepared with LigPrep (Schrödinger Maestro) at physiological pH. Docking was performed with Schrödinger Glide SP. The grid box was centered on the predicted binding pocket of the MyD88 TIR domain, defined from literature-reported key functional residues and validated by SiteMap cavity prediction, with box dimensions sufficient to enclose the whole pocket. The protocol was validated by re-docking the reference ligand, which reproduced the experimental binding pose with an RMSD <2.0 Å. For MD simulation, the KA–MyD88 complex was built in Desmond, solvated with water and ions, energy-minimized, and subjected to a single 100-ns simulation at 300.0 K and 1 atm (NPT). Trajectories were analyzed for protein/ligand RMSD, protein RMSF, and protein–ligand interactions. Because a single 100-ns trajectory was used, the conclusions should be interpreted with caution; replicate runs are planned for future studies.

### 2.10 Cellular thermal shift assay

TGF-β1-activated LX-2 cells were treated with either KA (10 μM) or DMSO for 3 h. Following incubation, cells were harvested and allocated into groups for subsequent temperature-based experiments. A series of temperatures, including 37 °C, 41 °C, 45 °C, 49 °C, 53 °C, 57 °C and a constant 55 °C, was set. Each group of cells was then subjected to these specific temperature treatments. Specifically, the cells were heated in a PCR machine for 3 min, left to equilibrate at room temperature for 3 min, and immediately subjected to repeated freeze-thaw cycles in liquid nitrogen. Following the temperature-related treatment, cells were lysed and centrifuged at 20,000 × g for 20 min to collect the supernatant. Subsequently, 30 μL of 5× loading buffer was added to the collected supernatant; the resultant mixture was boiled at 90 °C for 10 min to prepare samples for loading. Finally, all subsequent immunoblotting procedures were performed as previously reported (Lin et al., 2023). Exposure was then carried out to detect changes in protein levels at different temperatures.

### 2.11 MyD88 target knockdown and overexpression

Healthy log-phase LX-2 cells were seeded in 6-well plates and cultured at 37 °C with 5% CO_2_. Transfection was initiated when cell confluence reached 50%–60%. For MyD88 knockdown, MyD88 shRNA and Lipofectamine 3000 were mixed in an RNase-free tube following the manufacturer’s instructions (Shanghai GeneChem Co., Ltd., Shanghai, China). The mixture was incubated at room temperature for 20 min before being added to the cells. For MyD88 overexpression, pcDNA3.1-MyD88 plasmid and Lipofectamine 3000 were mixed under nuclease-free conditions, incubated for 20 min at room temperature, and applied to cells. Ten hours post-transfection, the culture medium was discarded; cells were rinsed with pre-warmed 37 °C PBS and supplied with fresh complete medium. Cells were further incubated for 48 h. Subsequently, cells were treated with 10 μM KA for 24 h, and Western blot analysis was performed to evaluate the knockdown and overexpression efficiency.

### 2.12 Statistical analysis

All statistical calculations and figure plotting were completed via GraphPad Prism software. Numerical results are presented as mean ± SD. Normality was assessed by the Shapiro–Wilk test and homogeneity of variances by Levene’s test; all datasets met the assumptions for parametric testing. Differences among ≥3 groups were evaluated by one-way ANOVA followed by Tukey’s multiple comparison test; when multiple treatment groups were compared with a single control, Dunnett’s test was applied. Two-group comparisons (e.g., Figure 5A) used the unpaired two-tailed Student’s t-test. CETSA thermal-shift data were analyzed by two-way ANOVA followed by a *post hoc* test. Differences were considered significant at p < 0.05, and exact p-values are reported in the text and figures. Biological replicates (n) denote independent experiments; technical replicates were averaged before analysis.

## 3 Results

### 3.1 The EAF fraction of *Sophora flavescens* exerts notable anti-liver fibrosis effects

The hepatoprotective activity of the ethyl acetate fraction (EAF) extracted from total flavonoids of *S. flavescens* was assessed in a CCl_4_-triggered hepatic fibrosis mouse model using multiple complementary assays. These included H&E staining to examine liver histopathology, serum AST and ALT measurements, Masson’s trichrome staining to visualize collagen accumulation, hepatic hydroxyproline (Hyp) quantification, as well as Western blot quantification for the expression levels of α-SMA and fibronectin proteins. The key findings are presented below.

For hepatic tissue pathology via H&E staining (top panels of Figure 1A), liver sections from normal controls displayed well-preserved architecture, orderly arranged hepatocytes, and no observable inflammation or necrosis. In contrast, the model group showed marked inflammatory infiltration, widespread hepatocyte injury, and disrupted lobular structure. Compared to the model, both the positive-control (silibinin, 100 mg/kg) and extract-treated groups had alleviated lesions, reduced inflammation, and less hepatocyte necrosis, with the high-dose EAF (100 mg/kg) group showing the most significant improvement. For Masson staining of hepatic sections (bottom panels of Figure 1A), in the normal control, minimal collagen fibers were present in the liver, mainly in the central vein and portal area. In the model group, there was a large-scale proliferation of collagen fibers, disrupting the lobule structure. In contrast, both the positive control and extract-treated groups exhibited markedly attenuated collagen accumulation compared to the model (P < 0.01), with the high-dose EAF group showing the most pronounced reduction (P < 0.01) Quantitative image analysis of Masson-stained sections using ImageJ (collagen-positive area as a percentage of the total section area) confirmed these histological findings: compared with the model group, the collagen-positive area was significantly reduced in the silibinin- and EAF-treated groups in a dose-dependent manner, with the high-dose EAF (100 mg/kg) group showing the greatest reduction (Figure 1B; P < 0.05 and P < 0.01, respectively).

As shown in Figure 1B, compared with the normal control group, the levels of serum AST, ALT and liver hydroxyproline (Hyp) in the model group were significantly increased (*P < 0.01*). After treatment with positive drugs and EAF, the above indices were significantly decreased (*P < 0.05*) in a dose-dependent manner, and the improvement effect of the high-dose EAF group was the most significant (*P < 0.01*). For α-SMA and fibronectin (FN) protein expression (Figure 1C), both markers were upregulated in the model group (*P* < 0.01). In contrast, the positive-control and extract-treated groups showed dose-dependent reductions in α-SMA and FN expression (*P* < 0.05), with the high-dose extract producing the greatest effect (*P* < 0.01).

In summary, the EAF of total flavonoids from *S. flavescens* effectively attenuated CCl_4_-induced liver fibrosis in mice. It alleviates hepatic histopathological lesions, reduces serum AST and ALT activities, decreases collagen fiber deposition, and downregulates fibrosis-related marker proteins in a dose-dependent manner, with the most pronounced effects observed in the high-dose group.

### 3.2 Identifying the anti-liver fibrosis compounds in the EAF of total flavonoids from *Sophora flavescens*

To assess the cytotoxicity of the isolated compounds from *S. flavescens*, we treated LX-2 cells with 35 compounds (40 μM) and the EAF (25 μg/mL) without TGF-β1 stimulation. The results showed that most compounds exhibited no substantial cytotoxicity at 40 μM, with cell viability remaining above 80% (Figure 6A; Supplementary Table S1), indicating their good safety profile at the screening concentration. Then, TGF-β1 (10 ng/mL) was applied for 24 h to induce LX-2 cell proliferation, a well-established model for HSC activation *in vitro* (Supplementary Figure S1). As expected, TGF-β1 treatment significantly increased LX-2 cell viability. Both the EAF and several tested compounds suppressed TGF-β1-driven LX-2 cell growth by over 50%, without causing obvious cytotoxicity (Figure 6B; Supplementary Table S2). Using the MTS assay, 11 compounds exhibited supportive antiproliferative activity against TGF-β1-stimulated LX-2 cells, with half-maximal inhibitory concentration (IC_50_) values falling between 4 and 40 μM (Supplementary Figure S2). Among these, eight molecules—kurarinol A, kurarinol B, kushenol Q, sophoraflavanone B, kurarinol, kushenol A, sophoflavescenol, and noranhydroicaritin—have not been previously reported in this context.

**FIGURE 6:**
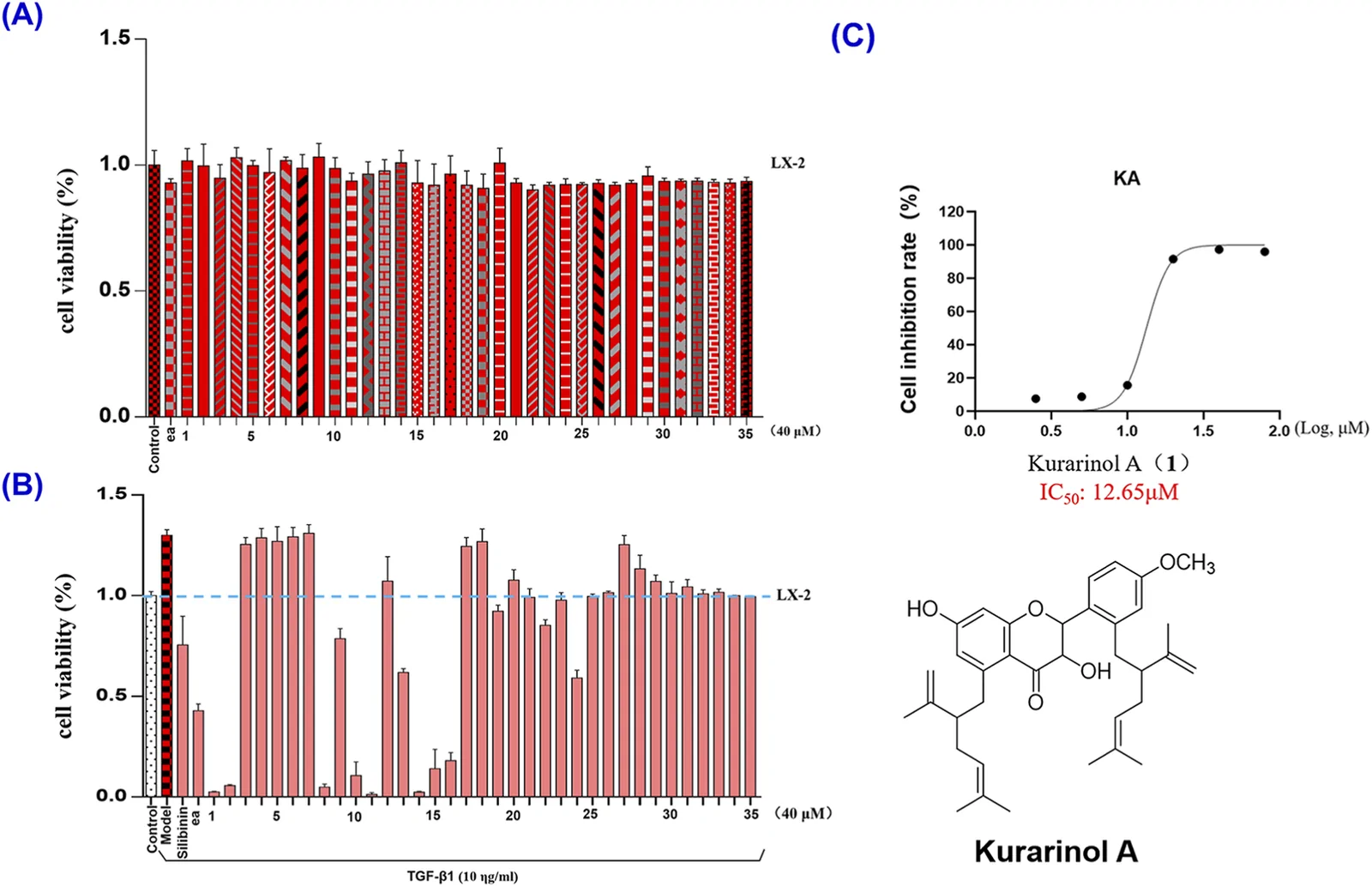
Cytotoxicity and anti-proliferative activity screening of 1–35 compounds in LX-2 cells. **(A)** The MTS assay was used to evaluate the cytotoxicity of compounds 1–35 against LX-2 cells. **(B)** The anti-proliferative activity of compounds 1–35 against TGF-β1-activated LX-2 cells was evaluated by the MTS assay, with silibinin (40 µM) as the positive control. Data are mean ± SD (n = 3), expressed as a percentage of control. **(C)** Dose-response curve of kurarinol A (KA) for inhibiting TGF-β1-activated LX-2 cells, showing an IC_50_ value of 12.65 μM. The chemical structure of KA is also presented.

The EAF (25 μg/mL) reduced TGF-β1-stimulated LX-2 cell viability to below 50%. Most of the purified compounds likewise inhibited LX-2 cell growth. Among the 11 active compounds, KA (Compound 1) exhibited an IC_50_ of 12.65 µM, ranking third of the 11 hits in potency (range: 4.80–40.35 µM; dose–response curves and individual IC_50_ values of all 11 active compounds are shown in Supplementary Figure S2) (Figure 6C). KA was prioritized for detailed mechanistic follow-up for two reasons. First, it is a rare lavandulyl-type flavanone whose unique chemical scaffold is structurally distinct from the other active compounds, making it of particular interest for target exploration. Second, our previous studies indicated that KA can modulate inflammatory signaling in hepatic cells (Yang et al., 2025; Lin et al., 2023), providing prior in-house evidence that supported its prioritization for functional investigation. Western blot analysis further showed that both the EAF and KA downregulated FN expression, with KA at 5–10 µM showing greater efficacy than the extract at 15–30 μg/mL (Supplementary Figure S3). This indicates a clear link between the bioactivity of the EAF and the efficacy of its individual components. Thus, further studies on the anti-fibrotic potential of KA from the EAF of *S. flavescens* are warranted for developing small-molecule therapeutics against liver fibrosis.

### 3.3 KA inhibited the proliferation and migration and facilitated apoptosis of activated LX-2 cells

Based on the IC_50_ value of 12.65 μM for KA, 0–40 μM was tested to determine appropriate doses for further experiments. Under TGF-β1 stimulation, fibronectin mRNA expression in LX-2 cells was quantified via RT-qPCR. Based on the results, KA concentrations of 2.5, 5, and 10 µM were chosen for further investigation. In TGF-β1-activated LX-2 cells, KA inhibited cell viability in a dose-dependent manner and reduced fibronectin mRNA expression (Figure 7A). The scratch test showed that KA suppressed cell migration at 24 and 48 h post-scratch (Figure 7B). Flow cytometry results showed that the proportion of apoptotic cells in the KA treatment group increased from 10.3% (2.5 μM) to 19.9% (10 μM), the model control group was 6.8%, and the silibinin group was 13.6% (Figure 7C). These findings demonstrate that KA induces apoptosis in LX-2 cells, thus decreasing the population of activated hepatic stellate cells. Therefore, KA inhibited the proliferation, suppressed the migration, and induced apoptosis of activated LX-2 cells.

**FIGURE 7:**
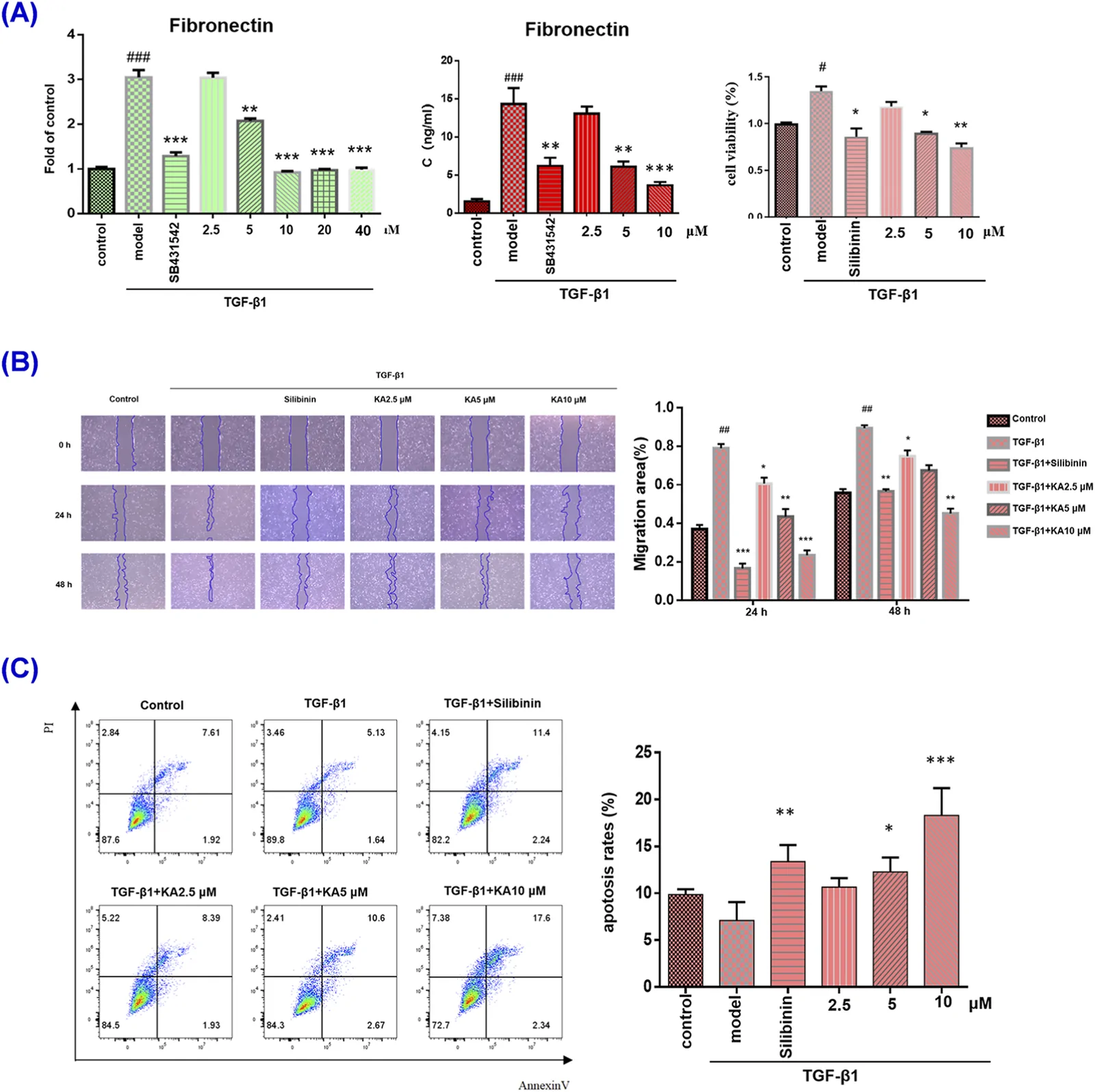
KA inhibits proliferation, migration and promotes apoptosis of TGF-β1-induced LX-2 cells. **(A)** Effects of varying concentrations of KA on the proliferation and fibronectin mRNA expression of TGF-β1-stimulated LX-2 cells. **(B)** Cell migration was observed at 0, 24, and 48 h after KA treatment (100×). The relative migration rate was calculated to quantify cell motility. Data are presented as mean ± SD from three independent experiments. **(C)** Apoptosis of activated LX-2 cells was detected by Annexin V-FITC/PI double staining and flow cytometry (left); the total apoptosis rate (Annexin V^+^ cells) was quantified (right). For all panels, data are presented as mean ± SD (n = 3 independent experiments). Statistical significance was determined by one-way ANOVA followed by Tukey’s *post hoc* test: ^#^ *P* < 0.05, ^##^ *P* < 0.01, ^###^ *P* < 0.001 vs. control; *P < 0.05, **P < 0.01, ***P < 0.001 vs. TGF-β1 model group.

### 3.4 KA inhibited the expression of fibrosis biomarkers fibronectin, α-SMA and collagen I in TGF-β1-stimulated LX-2 cells

After stimulation with TGF-β1, LX-2 cells were treated with various concentrations of KA (2.5, 5, 10 μM) for 48 h. The results showed that mRNA levels of fibronectin, α-SMA, and collagen I induced by TGF-β1 were significantly reduced (Figure 2A). Consistent with the trend of mRNA change, KA decreased the expression of the above proteins in a concentration-dependent manner (Figure 2B). These results collectively show that KA reduces the expression of fibrotic markers in TGF-β1-activated LX-2 cells.

### 3.5 Transcriptomic exploration of the mechanisms by which KA suppresses LX-2 cell activation

Principal Component Analysis (PCA) revealed clear separation in gene expression profiles across the control, model, and KA-treated groups, confirming distinct transcriptomic signatures (Figure 8A). Compared with the control group, TGF-β1 stimulation upregulated 444 genes and downregulated 2,947 genes (Figure 8B). Relative to the model group, KA treatment modulated 216 upregulated and 119 downregulated genes; importantly, KA reversed only a modest and specific subset of the TGF-β1-regulated transcriptome (Figures 8B,C). For GO and KEGG pathway enrichment analysis of the 34 overlapping target genes, all detected expressed genes in LX-2 transcriptome served as the background gene set. The Benjamini–Hochberg method was applied for multiple-testing correction, and terms with adjusted p-value <0.05 were considered significantly enriched. We note that the input gene list (n = 34) is relatively small, and enrichment results should be interpreted with caution. The heatmap illustrates the dynamic expression patterns of these genes among the normal, model, and KA groups, which were upregulated upon TGF-β1 stimulation and downregulated again after KA treatment (Figure 8D). KEGG pathway analysis linked these DEGs to multiple signaling pathways, including vascular smooth muscle contraction, cholesterol metabolism, cardiac muscle contraction, and the Toll-like receptor (TLR) signaling pathway (Figure 8E). Gene Ontology (GO) analysis revealed that these DEGs are potentially associated with multiple biological processes (BP), cellular components (CC), and molecular functions (MF) (Figure 8F).

**FIGURE 8:**
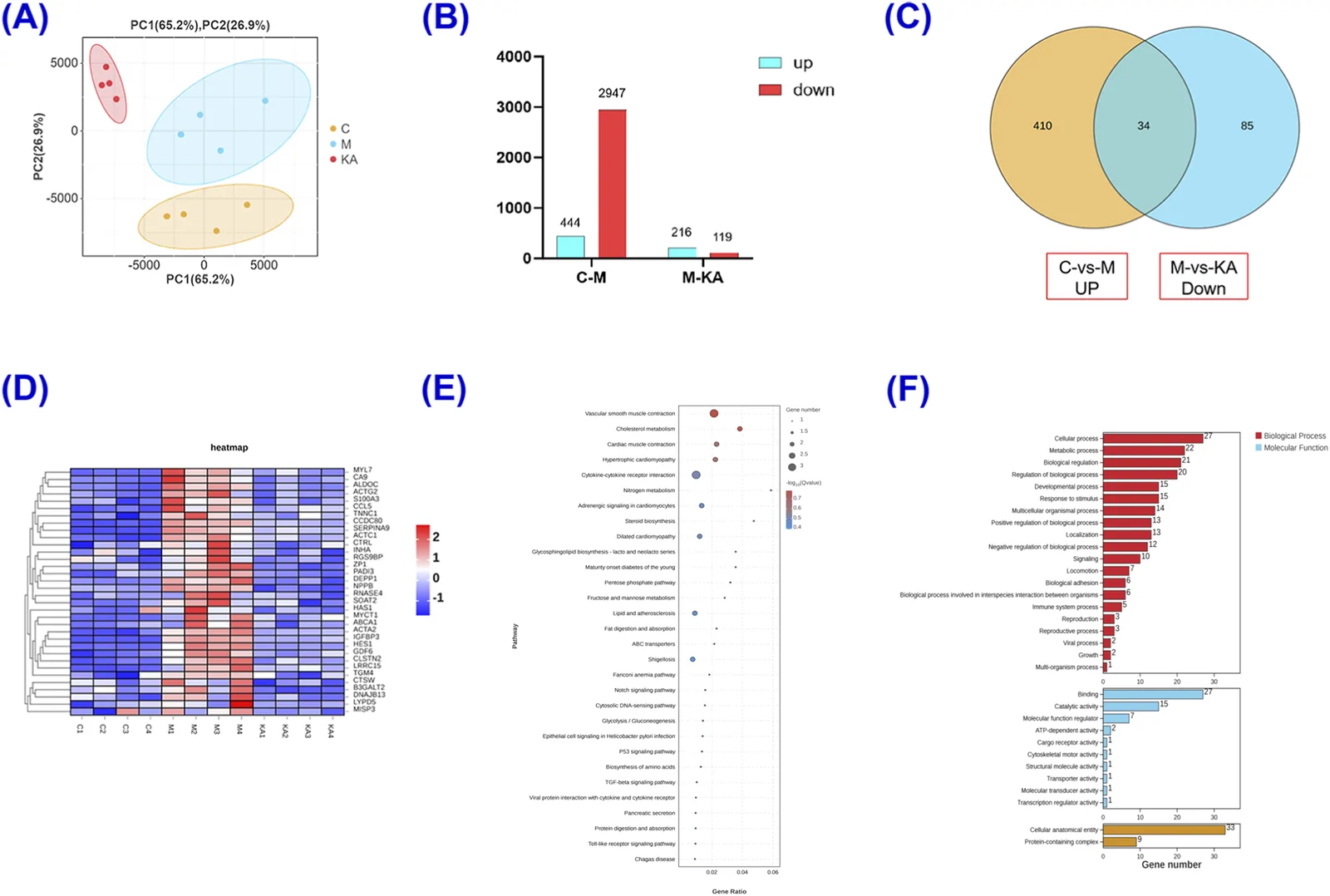
Transcriptome analysis. **(A)** PCA analysis. Each point represents a biological replicate; samples clustered together exhibit high similarity, whereas those farther apart show greater divergence. (C: control; M: model; KA: KA-treated) **(B)** Numbers of upregulated and downregulated genes in control vs. model and KA vs. model comparisons. **(C)** Venn diagram of DEGs from control, model and KA groups. **(D)** Heatmap of DEG expression profiles. Rows represent individual genes, and columns represent samples; red: upregulated, blue: downregulated. **(E)** KEGG pathway enrichment analysis. Dot color indicates significance (P-value); dot size reflects gene count. **(F)** GO enrichment analysis. They show biological process (green), cellular component (pink), and molecular function (yellow) terms. High-resolution versions of Panels E and F are available in Supplementary Figure S1.

### 3.6 KA inhibits LX-2 cells activation via the TLR4/NF-*κ*B/MyD88 inflammatory signaling pathway

Compared with the untreated control, LPS (100 ng/mL) stimulation significantly increased the mRNA levels of toll-like receptor 4 (TLR4), myeloid differentiation factor 88 (MyD88), Toll/IL-1R Domain-Containing Adaptor Protein (TIRAP), tumor necrosis factor receptor-associated factor 6 (TRAF6), transforming growth factor-β-activated kinase 1 (TAK1), nuclear factor kappa-B (NF-*κ*B), interleukin-1β (IL-1β) and Tumor Necrosis Factor-α (TNF-α) in LX-2 cells; these increases were dose-dependently reduced by KA (2.5, 5, 10 μM) (Figure 3A). Consistent with the RT-qPCR data, Western blotting confirmed similar trends at the protein level (Figure 3B).

### 3.7 KA demonstrates favorable molecular docking with MyD88 and maintains relatively stable binding during molecular dynamics simulations

Molecular docking simulation results showed that the binding energy between KA and MyD88 was −8.573 kcal/mol (Figure 9A). This low docking score supports a favorable binding pose within the predicted pocket and is consistent with the stable KA–MyD88 complex observed in the MD simulation. Further analysis of the binding site revealed three hydrogen-bonding interactions and ten hydrophobic interaction sites. The hydrogen-bonding interactions involved Arg251, Leu252, and Phe270 of MyD88 (detailed parameters are provided in Supplementary Table S3). These hydrogen bonds are mediated by the protein main-chain (backbone) amide and carbonyl groups of Arg251, Leu252, and Phe270, rather than by their side chains. The hydrophobic interactions were contributed by residues including Leu241, Lys250, Ile253 (three contacts), Phe270, Ile271 (two contacts), Ala292, and Leu295 (detailed parameters are provided in Supplementary Table S4). In a 100 ns molecular dynamics simulation (Figure 9B), analysis of the protein-ligand root-mean-square deviation (RMSD) showed initial fluctuations during the first 20 ns, after which both RMSD values reached a plateau, indicating that the complex attained a stable state and that KA binding did not induce significant conformational changes in the overall protein structure. The ligand RMSD (both Lig fit Prot and Lig fit Lig) further confirmed that KA remained steadily bound within the binding pocket of MyD88, without notable displacement or internal atomic fluctuations. Protein root-mean-square fluctuation (RMSF) analysis revealed higher flexibility at the N- and C-termini, while the α-helix and β-sheet regions exhibited greater rigidity; the loop regions displayed intermediate flexibility. These observations are consistent with typical protein dynamics and suggest that KA binding did not abnormally affect the structural dynamics of MyD88. The protein-ligand interaction analysis indicated that hydrogen bonds, hydrophobic contacts, ionic bonds, and water bridges persisted and remained stable throughout the simulation. Key residues such as ARG_251, LEU_252, and SER_244 contributed to maintaining complex stability, with these contacts persisting for more than 30% of the simulation time (Figure 9B).

**FIGURE 9:**
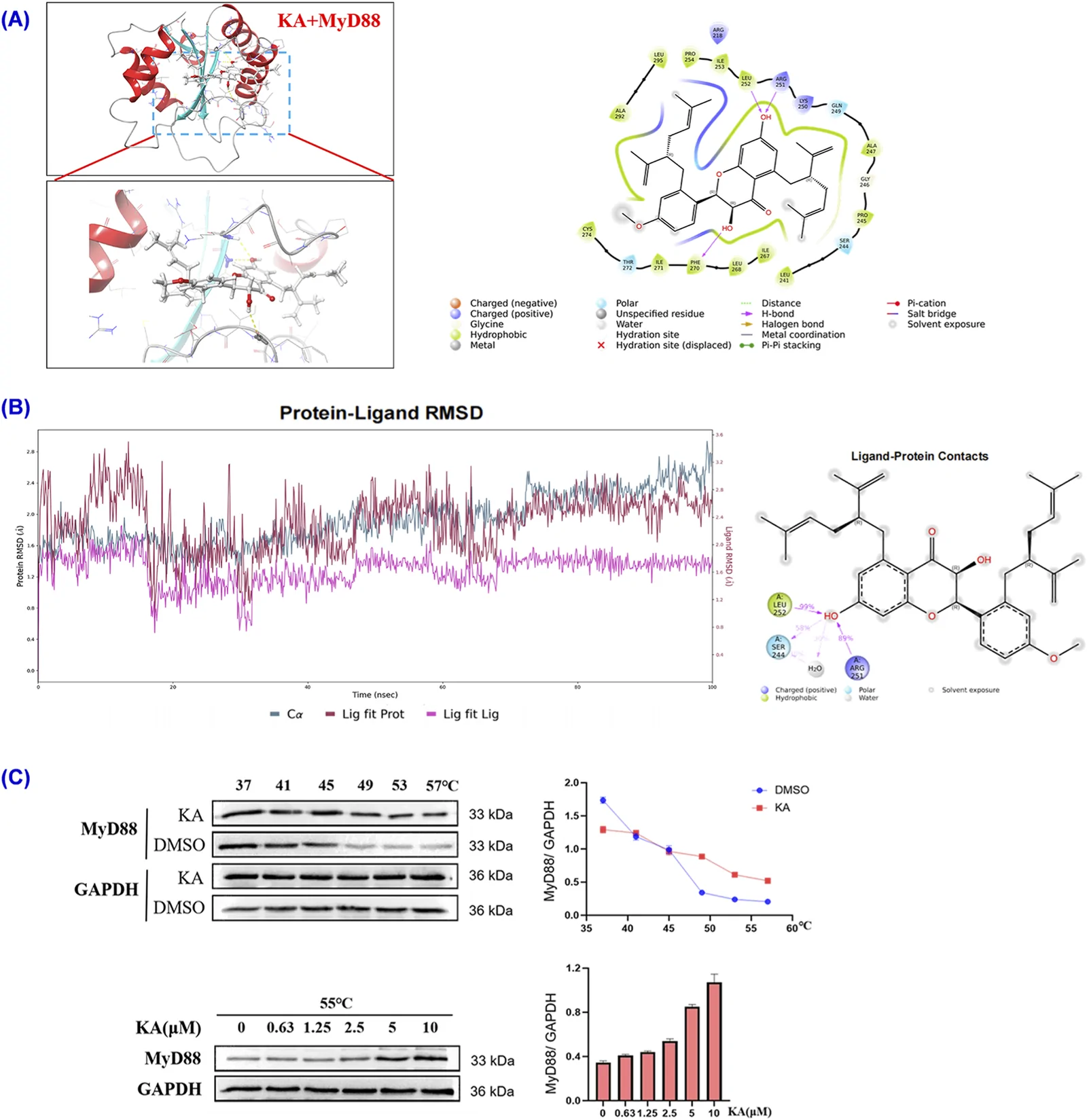
Molecular interactions and thermal stability of the KA-MyD88 complex. **(A)** KA and MyD88 molecular docking 3D map and 2D diagram. **(B)** Molecular dynamics (MD) simulation of the KA-MyD88 complex. Protein-ligand root-mean-square deviation (RMSD) curves over 100 ns simulation: cyan denotes protein Cα backbone RMSD; red denotes KA RMSD (relative to the protein); magenta denotes KA RMSD (relative to its initial conformation), with axes representing simulation time (ns) and RMSD (Å). Ligand-protein contact map illustrating interactions between KA and MyD88 residues (LEU252, SER244, ARG251), alongside corresponding interaction types (hydrophobic, polar) and their occurrence frequencies. **(C)** CETSA analysis of the KA–MyD88 interaction. Top: thermal stability of MyD88 in LX-2 cells treated with KA (10 µM) or DMSO across a temperature gradient of 37 °C–57 °C. Bottom: concentration-dependent stabilization of MyD88 by KA (0, 0.63, 1.25, 2.5, 5, and 10 µM) at a fixed temperature of 55 °C.

### 3.8 The binding of KA to MyD88 can significantly enhance the thermal stability of the target protein MyD88

CETSA revealed that MyD88 in the KA-treated group exhibited higher thermal stability than that in the DMSO control group across temperatures ranging from 37 °C to 57 °C. At 55 °C, KA enhanced MyD88 thermal stability in a concentration-dependent manner (Figure 9C).

### 3.9 MyD88 functions as the core drug target of KA in the context of anti-liver fibrosis

While the previous section identified KA as a potential MyD88 inhibitor, it remains unclear whether MyD88 serves as the drug target through which KA inhibits LX-2 cell activation and alleviates liver fibrosis. Accordingly, we designed a series of experiments centered on validating whether MyD88 is the key target responsible for KA’s anti-fibrotic effects in activated LX-2 cells.

In the MyD88 knockdown experiment, three MyD88-targeting virus strains (shMyD88-94, 95, 96) were selected for transfection of LX-2 cells. After rigorous screening, shMyD88-96 achieved the most substantial knockdown of MyD88 at the protein level (Figure 4A, with densitometric quantification shown in the corresponding bar graphs) and was therefore selected for the subsequent experiments. Subsequent in-depth analysis revealed that, compared with the model group (Figure 4B), knockdown of MyD88 significantly reduced liver fibrosis-related indicators. The expression of α-SMA, fibronectin, and collagen I was significantly downregulated, confirming MyD88 as a key mediator of profibrotic responses. More importantly, compared with the MyD88 knockdown alone group, KA treatment in MyD88-knockdown cells failed to further reduce the expression of liver fibrosis markers (α-SMA, fibronectin, and collagen I) or MyD88 downstream proteins (TAK1, p65, and TNF-α), strongly suggesting that KA exerts its anti-fibrotic effect through targeting MyD88. Consequently, when MyD88 is knocked down, the downstream pathway required for KA’s action is disrupted, thereby abolishing its ability to further inhibit fibrosis-related indicators.

For the MyD88 overexpression experiment, successful transfection of MyD88 was first confirmed, ensuring experimental reliability. Subsequent analyses showed that elevating MyD88 levels strongly promoted fibrotic responses, with clear increases in α-SMA, fibronectin, and collagen I (Figure 5A). In contrast, when KA was added to cells with MyD88 overexpression, this pro-fibrotic effect was markedly reduced, as seen by lowered expression of α-SMA, fibronectin, and collagen I (Figure 5B). These findings lend additional support to the idea that MyD88 serves as a possible drug target for KA in alleviating liver fibrosis, demonstrating that KA remains effective even when fibrosis is aggravated by MyD88 overexpression, thereby lessening the extent of liver fibrosis.

In conclusion, the knockdown and overexpression experiments indicate that MyD88 is a central target through which KA suppresses activated LX-2 cells and exerts anti-fibrotic activity. This provides a solid experimental foundation for future studies on KA’s anti-fibrotic mechanism and for designing MyD88-directed therapeutic strategies against liver fibrosis.

## 4 Discussion

In this study, we identified kurarinol A (KA) as a candidate MyD88 inhibitor, with convergent evidence from CETSA, computational predictions, and functional knockdown supporting MyD88 engagement, and systematically elucidated its molecular mechanism of inhibiting TLR4/NF-*κ*B signaling pathway by targeting MyD88 to exert anti-inflammatory and anti-fibrotic effects. This finding not only provides a modern pharmacological explanation for the traditional hepatoprotective application of *S*. *flavescens*, but also provides a promising candidate warranting lead optimization for the treatment of liver fibrosis targeting MyD88.

### 4.1 The interaction of KA with MyD88 and its pharmacological significance

TLR4/NF-*κ*B signaling pathway plays a key role in the inflammatory response of liver fibrosis (Jagavelu et al., 2010). As a key adaptor molecule downstream of TLR4, MyD88 is located at the convergence point of multiple inflammatory signaling pathways and regulates the expression of a series of inflammation-related genes. Thus, targeting MyD88 may achieve more efficient pathway blockade than targeting upstream receptors or downstream kinases (O'Neill and Bowie, 2007). However, selective MyD88 small-molecule inhibitors are still lacking, and TJ-M2010-5, which has entered clinical trial, also has limitations such as poor druggability (Xie et al., 2022). KEGG enrichment analysis identified the TLR pathway as one of the enriched cascades following KA treatment, suggesting that the pathway may mediate the anti-fibrotic effects of KA.

By integrating transcriptome analysis, molecular dynamics simulation and CETSA, this study obtained evidence consistent with a direct interaction between KA and MyD88 that modulates the TLR4/NF-*κ*B signaling pathway. Transcriptome data showed that KA treatment significantly enriched the expression changes of genes related to the NF-*κ*B/TLR signaling pathway, which was highly consistent with the characteristics of the MyD88-deficient transcriptome reported by (16). Molecular docking and dynamic simulation further revealed the binding mode of KA and MyD88 at the atomic level. KA stably binds to the TIR domain of MyD88 through supportive hydrogen bonds and hydrophobic interactions, and the binding free energy is −8.573 kcal/mol, indicating that there is a thermodynamically favorable binding between the two. A 100 ns molecular dynamics simulation showed that the KA-MyD88 complex the protein Cα RMSD stabilized at approximately 1–2 Å after the initial equilibration (∼20 ns) with no systematic drift; the ligand-fit-to-protein RMSD fluctuated within ∼1–3 Å, reflecting conformational adjustment of the flexible lavandulyl side chain rather than ligand dissociation, and the ligand internal RMSD remained low and stable (∼0.5–1 Å). Post-equilibrium conformational fluctuations of the complex were below 1.5 Å, further supporting the pharmacological relevance of this binding event. These computational findings provide a clear direction for future validation of the key binding residues through site-directed mutagenesis (O'Neill and Bowie, 2007; Song et al., 2024). CETSA confirmed the direct binding of KA to MyD88 in a dose-dependent manner. KA treatment improved the thermal stability of MyD88, suggesting that KA may block the activation of downstream NF-*κ*B by stabilizing MyD88 in a non-productive conformation, a hypothesis to be tested and preventing it from being recruited by TLR4 (Kasai et al., 2026). Taken together, these observations indicate that such regulation likely represents a key mechanism by which KA counteracts hepatic fibrosis.

### 4.2 The dual anti-inflammatory and anti-fibrotic effects of KA

In addition to its effect on the TLR4/MyD88/NF-*κ*B axis, our previous work (Yang et al., 2025) showed that KA also inhibits TGF-β/Smad signaling. TGF-β, as a core profibrotic factor, activates HSCs and promotes ECM deposition through Smad-dependent and Smad-independent pathways (Hernandez-Gea and Friedman, 2011). Because MyD88 and TGF-β signaling converge on HSC activation in chronic liver disease (Fu et al., 2025), the concurrent modulation of both pathways by KA may be particularly relevant to its anti-fibrotic activity. Whether KA engages multiple fibrosis-related targets simultaneously remains to be determined; this hypothesis will be tested in future studies (Wenbo et al., 2024; Ciceu et al., 2024).

### 4.3 Genetic validation and target identification

Through gene knockdown and overexpression experiments, this study further ssupports that MyD88 is a functional target for KA to exert anti-fibrotic effects. Knockdown of MyD88 eliminated the anti-fibrotic effect of KA, whereas in MyD88-overexpressing cellsKA reversed MyD88-driven fibrosis. These results are consistent with an important role of MyD88 in mediating the anti-fibrotic effects of KA. Because MyD88 serves as a universal adaptor for multiple TLR/IL-1R family members (Chen et al., 2020; Janssens and Beyaert, 2002), whether KA affects other MyD88-dependent pathways (e.g., IL-1R signaling) beyond TLR4 remains to be investigated.

### 4.4 Comparison with previous studies

Previous studies have confirmed that KA has a protective effect on CCl_4_-induced acute liver injury, and its mechanism may be related to the inhibition of TLR2/NF-*κ*B signaling pathway (Lin et al., 2023). On this basis, this study further expanded the pharmacological activity of KA from acute liver injury to liver fibrosis model, and expanded from TLR2 signaling to the TLR4/MyD88/NF-*κ*B signal axis, which systematically revealed the molecular mechanism of KA targeting MyD88 for the first time. This finding provides a more accurate target and a more complete mechanism explanation for the anti-fibrosis effect of KA.

### 4.5 Research limitations

Although this study provides supportive evidence, it still has some limitations. First, the *in vivo* pharmacodynamic data of this study were from the ethyl acetate active fraction, rather than KA pure product. Although *in vitro* and computational data support a direct interaction between KA and MyD88, the systematic pharmacokinetic-pharmacodynamic study of the KA pure product still needs to be carried out independently in the follow-up work. Second, the model induced by CCl_4_ for 4 weeks belongs to the acute-subacute liver injury model, which has not yet covered the spontaneous regression of liver fibrosis or the progression stage of cirrhosis (Ipsen et al., 2020). Third, the key amino acid residues of the interaction between KA and MyD88 still need to be further confirmed by site-directed mutagenesis experiments. Fourthly, several quantitative analyses, including Western blot densitometry and CETSA, were based on only three independent biological replicates (n = 3), which limits the statistical power to detect smaller differences; studies with larger sample sizes are warranted to consolidate these findings. Fifthly, the pharmacokinetic properties of KA, such as absorption, distribution, metabolism and excretion, have not been fully elucidated. In this regard, hepatic exposure of KA following oral administration of the EAF (e.g., hepatic Cmax and AUC) was not estimated, and whether the concentrations shown to be effective *in vitro* (2.5–10 μM) are achievable in the target organ *in vivo* remains to be established; quantifying liver KA exposure should therefore be a priority in future pharmacokinetic studies. Sixthly, the present study used only male mice, and whether KA and its EAF exert comparable anti-fibrotic effects in female mice and in sex-diverse populations remains to be determined; evaluation across both sexes should be included in future studies. We acknowledge that the present data support a direct interaction of KA with MyD88 but do not establish selectivity, as systematic profiling against other TIR-domain-containing adaptors (e.g., TIRAP, TRIF, TRAM) or TLR4 itself was not performed; such selectivity assessment is a required future study. In addition, a dedicated quantitative HPLC purity determination of the KA batch used here was not performed; KA identity was confirmed by comparison of its spectroscopic data with the literature (Li et al., 2021), and quantitative purity assessment of the isolated compound is planned in future work. Finally, although KA is structurally distinct from canonical pan-assay interference (PAINS) scaffolds and its effects are dose-dependent, concentration-dependent (CETSA), and abolished by MyD88 knockdown, dedicated PAINS-exclusion counter-screens (e.g., detergent-based aggregation assays and reducing-agent assays) and a direct biophysical binding assay (MST/SPR/ITC) were not performed; these are required to fully exclude non-specific interference and are listed as priorities for future work.

### 4.6 Conclusion

In conclusion, this study bridges traditional Chinese medicine and modern molecular pharmacology by integrating transcriptomics, computational biology, and functional genomics to elucidate the anti-fibrotic mechanism of KA. We identify MyD88 as a candidate molecular target and demonstrate that KA suppresses TLR4/NF-*κ*B signaling in a MyD88-dependent manner. Beyond deepening the mechanistic understanding of KA, this work provides a referential paradigm for systematic natural product research. However, translating KA into clinical practice will require further pharmacokinetic optimization, efficacy validation in diverse liver fibrosis models, and structure-based modification to enhance oral bioavailability and target selectivity.

## Data Availability

The RNA-seq datasets presented in this study have been deposited in NCBI BioProject under accession number PRJNA1542353. All other raw data supporting the conclusions of this article are available from the corresponding author upon reasonable request, without undue reservation.
