# Supplementary material for: Kurarinol A from Sophora flavescens suppresses hepatic stellate cell activation via the TLR4/MyD88/NF-κB pathway
Source: Front Pharmacol. 2026 Oct 22;17:1948257. doi: 10.3389/fphar.2026.1948257 (PMC13736048; doi:10.3389/fphar.2026.1948257)
Supplement: Supplementary file 1 [file Supplementaryfile1.docx]

**Supporting information**

**List of contents**

**Fig. S1** The optimal concentration and time of LX-2 cells in TGF-β1 modeling were screened.

**Fig. S2** The dose-cell viability curves for active compounds and their IC50 values.

**Fig. S3** A comparison of the inhibitory effects of ethyl acetate (EA) and kurarinol A (KA) on the expression levels of the fibrotic cytokine fibronectin.

**Fig. S4** Transcriptome analysis. Correlation thermogram of sample analysis of DEGs in different groups

**Fig. S5** Structural characterization of kurarinol A (KA): IR, UV, and HRMS spectra. Reproduced from Li et al., 2021

**Fig. S6** 1H NMR and 13C NMR spectra of kurarinol A (KA) (sample KS-34A). Reproduced from Li et al., 2021

**Table S1** The cytotoxic activities of compounds 1-35 on LX-2 cells.

**Table S2** MTS assay was used to screen the active compounds from compounds 1- 35 that inhibit the proliferation of LX-2 cells.

**Table S3** The hydrogen-bond binding sites of KA and MyD88

**Table S4** Hydrophobic interactions between KA and MyD88

**Table S5** The shRNA target sequences for shMyD88-94/95/96

Fig. S1 The optimal concentration and time of LX-2 cells in TGF-β1 modeling were screened.

Fig. S2 The dose-cell viability curves for active compounds and their IC50 values.

Fig. S3 A comparison of the inhibitory effects of ethyl acetate (EA) and kurarinol A (KA) on the expression levels of the fibrotic cytokine fibronectin. (^###^P < 0.001 compared with control; ^*^P < 0.1, ^**^P < 0.01, and ***P < 0.001 compared with the TGF-β1 model group)

Fig. S4 Transcriptome analysis. Correlation thermogram of sample analysis of DEGs in different groups.

Fig. S5 Structural characterization of kurarinol A (KA). **(A)** IR spectrum. **(B)** UV spectrum. **(C)** HRMS (ESI) spectrum. Reproduced from our previous study (Li et al., 2021) for the structural identification of kurarinol A.

Fig. S6 ¹H NMR (A) and ¹³C NMR (B) spectra of kurarinol A (KA) (sample KS-34A). The compound structure with atom numbering is shown in each panel. Reproduced from our previous study (Li et al., 2021) for the structural identification of kurarinol A.

**Table S1** The cytotoxic activities of compounds 1-35 on LX-2 cells.

| No. | compounds | Cell viability (LX-2) | No. | compounds | Cell viability (LX-2) |
| --- | --- | --- | --- | --- | --- |
|  | ethyl acetate extract | 90.8%(0.5%) | **18** | 7,3'-di-O-methyl | 92.0%(4.7%) |
| **1** | kurarinol A | 101.7%(3.9%) | **19** | genistein | 90.9%(4.6%) |
| **2** | kurarinol B | 99.6%(3.1%) | **20** | calycosin | 99.4%(3.8%) |
| **3** | kurarinol C | 94.8%(4.4%) | **21** | formononetin | 90.7%(4.4%) |
| **4** | kushenol H | 106.8%(0.2%) | **22** | biochanin A | 86.1%(3.8%) |
| **5** | kushenol L | 99.8%(1.6%) | **23** | 5,4'-dihydroxyflavone | 92.0%(0.9%) |
| **6** | kuraridinol | 97.1%(2.6%) | **24** | luteolin | 90.1%(4.2%) |
| **7** | kuraridine | 101.8%(1.1%) | **25** | 7-hydroxycoumarin | 90.2%(3.3%) |
| **8** | xanthohumol | 98.8%(4.4%) | **26** | 7,8-dihydroxycoumarin | 90.6%(2.5%) |
| **9** | isoliquiritigenin | 103.2%(4.4%) | **27** | 4-methoxysalicylic acid | 89.2%(2.1%) |
| **10** | kushenol Q | 98.7%(3.5%) | **28** | b-resorcylic acid | 91.4%(2.0%) |
| **11** | sophoraflavanone B | 93.6%(2.6%) | **29** | 4-hydroxybenzoic acid | 95.6%(3.0%) |
| **12** | naringenin | 96.5%(3.9%) | **30** | sophoracarpan A | 87.9%(3.6%) |
| **13** | kurarinol | 97.7%(3.7%) | **31** | trifohrhizin-6'-monoacetate | 92.3%(1.5%) |
| **14** | kushenol A | 99.7%(4.0%) | **32** | trifohrhizin | 90.8%(2.9%) |
| **15** | sophoflavescenol | 92.9%(2.2%) | **33** | maackiain | 92.4%(1.1%) |
| **16** | noranhyoicaritin | 92.1%(2.8%) | **34** | (6S,6a*S*,11a*R*）-6α-Methoxy-pterocarpin | 91.5%(2.0%) |
| **17** | quercetin | 96.4%(1.9%) | **35** | L-maackiain | 92.1%(1.8%) |

**Table S2** MTS assay was used to screen the active compounds from 1- 35 that inhibit the proliferation of LX-2 cells.

| No. | compounds | Cell viability (LX-2) | No. | compounds | Cell viability (LX-2) |
| --- | --- | --- | --- | --- | --- |
|  | control | 100.0%(1.7%) | **18** | 7,3'-di-O-methyl | 126.9%(2.1%) |
|  | model | 130.0%(2.2%) | **19** | genistein | 92.3%(2.5%) |
|  | Silibinin | 75.6%(1.6%) | **20** | calycosin | 107.8%(3.1%) |
|  | ethyl acetate extract | 42.9%(2.7%) | **21** | formononetin | 99.2%(3.4%) |
| **1** | kurarinol A | 2.5%(0.2%) | **22** | biochanin A | 85.3%(2.2%) |
| **2** | kurarinol B | 5.7%(0.4%) | **23** | 5,4'-dihydroxyflavone | 97.8%(3.1%) |
| **3** | kurarinol C | 125.6%(2.8%) | **24** | luteolin | 59.1%(3.2%) |
| **4** | kushenol H | 128.8%(3.8%) | **25** | 7-hydroxycoumarin | 99.7%(0.9%) |
| **5** | kushenol L | 127.0%(1.9%) | **26** | 7,8-dihydroxycoumarin | 101.6%(0.6%) |
| **6** | kuraridinol | 129.3%(3.8%) | **27** | 4-methoxysalicylic acid | 125.5%(3.6%) |
| **7** | kuraridine | 131.0%(3.5%) | **28** | b-resorcylic acid | 113.4%(2.5%) |
| **8** | xanthohumol | 4.9%(1.2%) | **29** | 4-hydroxybenzoic acid | 107.2%(2.6%) |
| **9** | isoliquiritigenin | 78.7%(3.0%) | **30** | sophoracarpan A | 101.2%(3.7%) |
| **10** | kushenol Q | 10.7%(2.5%) | **31** | trifohrhizin-6'-monoacetate | 104.4%(3.0%) |
| **11** | sophoraflavanone B | 1.4%(0.7%) | **32** | trifohrhizin | 100.9%(1.6%) |
| **12** | naringenin | 100.7%(2.7%) | **33** | maackiain | 101.7%(1.3%) |
| **13** | kurarinol | 68.7%(3.6%) | **34** | (6S,6a*S*,11a*R*）-6α-Methoxy-pterocarpin | 100.1%(0.1%) |
| **14** | kushenol A | 2.5%(0.3%) | **35** | L-maackiain | 99.6%(0.3%) |
| **15** | sophoflavescenol | 14.0%(3.9%) |  |  |  |
| **16** | noranhyoicaritin | 18.1%(3.2%) |  |  |  |
| **17** | quercetin | 124.5%(3.6%) |  |  |  |

**Table S3 The hydrogen bond binding sites of KA and MyD88**

| Index | Residue | Distance  AA  H-A | Distance  D-A | Donor  Angle | Donor  Atom | Acceptor  Atom | Bond type |
| --- | --- | --- | --- | --- | --- | --- | --- |
| 1 | 251A | ARG 2.46 | 3.09 | 121.36 | 772[Nam] | 42[O3] | backbone (main-chain N–H) |
| 2 | 252A | LEU 1.95 | 2.86 | 151.85 | 783[Nam] | 42[O3] | backbone (main-chain N–H) |
| 3 | 270A | PHE 2.00 | 2.83 | 139.56 | 30[O3] | 938[O2] | backbone (main-chain C=O) |

**Table S4 Hydrophobic interaction between KA and MyD88**

| Index | Residue | AA | Distance | Ligand Atom | Protein Atom |
| --- | --- | --- | --- | --- | --- |
| 1 | 241A | LEU | 3.47 | 41 | 707 |
| 2 | 250A | LYS | 3.24 | 26 | 769 |
| 3 | 253A | ILE | 3.45 | 29 | 798 |
| 4 | 253A | ILE | 3.62 | 23 | 797 |
| 5 | 253A | ILE | 3.78 | 24 | 796 |
| 6 | 270A | PHE | 3.52 | 40 | 939 |
| 7 | 271A | ILE | 3.74 | 6 | 953 |
| 8 | 271A | ILE | 3.49 | 1 | 951 |
| 9 | 292A | ALA | 3.70 | 28 | 1127 |
| 10 | 295A | LEU | 3.77 | 28 | 1148 |

**Table S5** The shRNA target sequences for shMyD88-94/95/96

| **NO.** | **Accession** | **Target Seq** | **CDS** | **GC%** |
| --- | --- | --- | --- | --- |
| MYD88-RNAi(120494-1) | NM_002468.5 | CATCAAGTACAAGGCAATGAA | 41..931 | 36.84% |
| MYD88-RNAi(120495-1) | NM_002468.5 | CCTGTCTCTGTTCTTGAACGT | 41..931 | 42.11% |
| MYD88-RNAi(120496-1) | NM_002468.5 | GCAAGGAATGTGACTTCCAGA | 41..931 | 47.62% |
| Description | Homo sapiens MYD88 innate immune signal transduction adaptor (MYD88), transcript variant 2, mRNA | | | |
